# Navigating racism, stigma, and autism services: A scoping review of the lived experiences of racially and ethnically minoritized families

**DOI:** 10.1371/journal.pmen.0000481

**Published:** 2025-11-13

**Authors:** Julia Sterman, Zoe Wagland, Louise Scott-Cole, Natasha Spassiani, Janet Njelesani

**Affiliations:** 1 Edinburgh Napier University, Edinburgh, United Kingdom; 2 Australian Catholic University, Watson, Australia; 3 Wake County, Cary, North Carolina, United States of America; 4 Department of Occupational Therapy, New York University, New York, New York, United States of America; National Psychological Association of Ukraine, UKRAINE

## Abstract

Research and clinical practice that addresses the needs of Autistic children often de-centres minoritized voices, despite the existing inequities that prevents their access to services and community participation. Grounded in Disability Critical Race Theory, this scoping review sought to collate and synthesise the research on the intersecting lived experiences of Autistic children and families from racially and ethnically minoritized backgrounds to inform more culturally attuned paediatric clinical practice. The authors systematically searched 8 databases up to June 2025. Extracted data from included articles were analysed using qualitative content analysis informed by Disability Critical Race Theory. Fifty-six studies were included in this scoping review, with a total of 1454 participants across the included studies. Findings illuminated that families had difficulty learning about and understanding autism, gaining access to services that met their cultural and language needs, and experienced disability-based stigma and racism. Families thrived when they were provided opportunities to learn about autism and available resources, could advocate for their child and others, access services from providers they trusted, and have their Autistic child celebrated within their community. To reduce inequities, there is a need for service providers to conduct culturally attuned paediatric clinical practice that centres the priorities of Autistic children and their families from racially and ethnically minoritized backgrounds. This paediatric practice needs to be neurodiversity-positive, culturally affirming, and financially, geographically, physically, socially, and culturally accessible.

## Introduction

In the last decade, there has been an important shift towards autism research and clinical practice that is neurodiversity-affirming and includes the voices of Autistic people, with the potential to improve the quality of life for Autistic people [1,2]. Some in the neurodiversity movement advocate for the acceptance of autism as an aspect of individual identity, viewing it as a difference rather than a deficiency [3], while others consider it a disability [4]. Due to the growing awareness that masking or camouflaging Autistic traits can lead to increased depression and suicide among Autistic people [5,6], there is a shift towards goals that focus on Autistic well-being, autonomy, and environmental goodness of fit rather than remediating perceived deficits [2,4]. Perceptions of the neurodiversity movement and autism are complex, in part due to misconceptions of the priorities of neurodiversity movement [4], differing perspectives on how to conceptualize autism and neurodiversity [7], and debates of whether the voices of Autistic adults or parents of Autistic children should be prioritized [8]. The current study aligns with the conceptualization of Ne’eman and Pellicano, in focusing on interventions that support Autistic individuals and their families across a range of support needs. These interventions focus on addressing areas that cause discomfort, distress, or risk while safeguarding the person’s autonomy, rather than attempting to fix non-harmful traits such as stimming.

Neurodiversity-affirming research has not been sufficiently inclusive of a range of ethnic and racial populations including Black, Asian, Indigenous, and minority ethnicity communities [9,10], immigrant families living in Global North countries [11], as well as families with non-Christian religious identities [12]. Because research has primarily been conducted with white Autistic children, there is a dearth of information about the lived experiences of racially and ethnically minoritized families of Autistic children.

From the existing research with racially and ethnically minoritized Autistic children, it is clear that significant disparities exist. Recent research has indicated a change in diagnosis patterns, with children from historically underrepresented groups for the first time having a higher autism diagnosis rate at 8 years old compared to white children [13]. However, despite the growing identification of children from historically underrepresented groups as Autistic, disparities persist in access to health services, referral frequency, and timely autism diagnosis [14,15]. In addition to disparities in diagnosis and service use, racially and ethnically minoritized Autistic populations also face disproportionate mental health challenges. Recent studies have documented elevated risks of anxiety, depression, and suicidality among Autistic individuals [16] and these risks are compounded for racially minoritized groups who experience intersecting racism and ableism in health and educational systems [17,18]}. Furthermore, parents of Autistic children from racially and ethnically minoritized backgrounds report unequal treatment in healthcare, discrimination while seeking and accessing services for their children, lower service hours, and poorer healthcare quality [15,19]. Promotion of access and participation is part of the Code of Ethics of many health and education practitioners [20] and as such it is vital that practice is directly informed by perspectives from racially and ethnically minoritized populations to appropriately serve all children.

To address the existing disparities, culturally attuned clinical and educational services could support racially and ethnically minoritized Autistic children and their families through responding to the family’s unique intersection between their culture and their family’s disability experience [21]. This is an important consideration, particularly given increasing racial, ethnic and cultural diversity within countries of the Global North. However, practice guidelines are often written and implemented without consideration of the intersectionality of a person’s race, ethnicity, religion, and neurodivergence [22]. This leads to inequitable access to an autism diagnosis and intervention for racially and ethnically minoritized Autistic children [9,10,23].

Currently, there is no existing published review that brings together perspectives of the lived experiences of racially and ethnically minoritized Autistic children and their families to understand their access to, right, and need for culturally attuned support. Previous reviews have focused on individual cultural groups, such as Māori [24] and Indigenous Australians [25], but none have reviewed the literature broadly to identify similarities and differences across racially and ethnically minoritized families with Autistic children to make recommendations for research and practice. The aim of this scoping review, therefore, is to collate and synthesise the research on the intersecting lived experiences of racially and ethnically minoritized Autistic children and their families. Secondly, this review will provide recommendations to enable providers working in health, disability, education, and social care sectors to practice in a culturally affirming manner when working with racially and ethnically minoritized Autistic children and their families living in the Global North.

### Theoretical framework

Disability Critical Race Theory offers a transformative lens through which to conduct and interpret scoping reviews, particularly in exploring the intersections of race, disability, and systemic inequities [26]. It highlights how intersecting identities contribute to layered and compounding forms of oppression and stigma (e.g., a Black Autistic individual is not only Black or only Autistic, but rather, navigates daily life as someone others perceive as Black and Autistic). In this review the theory not only helped to identify gaps in the literature but also how these gaps are shaped by systemic inequities. This theoretical lens enabled critique of systemic structures and resistance of deficit-based interpretations, ensuring that the research process was reflective of and responsive to the rights of Autistic communities. Furthermore, Disability Critical Race Theory was selected as the framework for this review because of its focus on centring lived experiences and ability to challenge ableist assumptions and biases.

### Positionality and language

Autistic communities value meaningful inclusion in research to maximize the potential for findings to be relevant to the intended communities (1). To ensure that the conclusions are neurodiversity-affirming and aligned with the Autistic community, we incorporated Autistic knowledge and lived experiences throughout every stage of this review. The research team was comprised of five members who identify as: (1) an English speaking, white and minority ethnicity female, immigrant, self-identifying as Autistic, occupational therapist, and academic with experience with scoping reviews (2) an English speaking, non-binary, Autistic white female, early career psychology researcher (3) an English speaking, white female, immigrant, ADHDer, and school based occupational therapist, with experience in scoping reviews (4) an English-speaking, white female, disabilities studies scholar, ADHDer with learning disabilities, and experience in community-based research for people with intellectual disabilities, (5) an English-speaking, non-disabled, white female, disability ally, occupational therapist, and mid-career academic with experience in scoping reviews.

Furthermore, Autistic expertise was sought in the form of an advisory board formed of Autistic adults. Some advisory board members also held the identities of being parents of Autistic children, and/or being from a racially and ethnically minoritized background. The advisory board informed the design of this scoping review and provided feedback on the findings and recommendations.

In recognizing the unique culture and experience of Autistic people, the authors chose to capitalize “Autistic” in alignment with the American Psychological Association’s guidelines for the Deaf and Blind communities [27] and People with Disabilities Australia’s guidance on language for Autistic people [28]. The authors recognize that there is no universal convention on whether to capitalize Autistic or not, and the preferences of the community are not homogenous, and evolve and change.

## Methods

The scoping review was conducted in accordance with the Joanna Briggs Institute (JBI) methodology for scoping reviews [29], with a protocol following the Preferred Reporting Items for Systematic Reviews and Meta-Analyses Guidance for Scoping Reviews (PRISMA-ScR) [30]. The review was not registered. The review protocol can be accessed by contacting the authors.

### Compliance with ethical standards

The authors have no conflicts of interest to declare in this research. This research did not involve data collection with human or animal participants; thus, no ethical permissions or informed consent was required.

### Eligibility criteria

Requirements of inclusion in this scoping study were studies that: a) were empirical and collected qualitative data, b) contained data about the experience of racially and ethnically minoritized Autistic children, aged from birth to 18 years old, and their families living in the Global North, c) were published in a peer-reviewed journal, and d) written in English or with a validated English translation regardless of the geographic origin of the research. Studies were excluded if they a) only included non-Hispanic white participants, b) did not report any qualitative perspectives from participants, c) were written in a language other than English with no validated translation. To align with the principles of Disability Critical Race Theory we excluded research that was solely focused on access to Applied Behavioural Analysis (ABA) services as these services perpetuate ableist approaches towards Autistic people [31], however, as ABA is very prevalent within autism services, we did not exclude studies that reported on families accessing ABA alongside discussions of others services and family experiences. To comprehensively address the research question through capturing family experiences, this scoping study focused on qualitative studies. Qualitative research is uniquely positioned to capture these nuances, offering insights that are often not obtainable through quantitative methodologies. No restrictions to inclusion were applied based on year of publication. Racially and ethnically minoritized families were determined to be participants living in the Global North (Western Europe, North America, New Zealand, and Australia) and identified as belonging to a racial or ethnic minority such as Black, Asian, Indigenous, Latino, Arab, or Traveller.

Inclusion and exclusion criteria were pilot tested on a subset of articles to ensure clarity, consistency, and applicability. This process reduced ambiguity and enhanced inter-rater reliability among reviewers across the review phases.

### Information sources and search

A systematic search of the peer-reviewed literature in eight databases (MEDLINE, PsycInfo, PsychArticles, PubMed, CINAHL, Scopus, Psychology and Behavioral Sciences Collection, and Web of Science) and was conducted in June 2025 using the search terms indicated in Table 1. The databases were chosen to comprehensively capture literature from sociology, medicine, psychology, health science, and disability studies. Reference lists of included articles were screened to identify further potential sources that would meet the inclusion criteria. We did not include grey literature in our search, as our focus was on peer-reviewed journal publications to gain insight into the current state of the scholarly field.

**Table 1:** Search strategy.

	Population	Area of interest
1	Immigration OR Immigrant OR Emigration OR Emigrant OR Migration OR Migrant OR Refugee	Immigrant perspectives
2	Minorit* OR Ethni* Minorit* OR Minorit* Perspect*	Minority perspectives
3	Cultural Influence OR Cultural Background OR Cross-Cultural OR Sociocultural Factors OR Multicultural* OR Cultural Identit*	Cultural influences
4	Global south OR Indigenous OR Black OR Native American OR aboriginal Australian OR Maori OR First Nation* OR Latin* OR Indian OR Pacific island* OR Chinese OR Nigeri* OR African OR Asia* OR South American OR Central American OR Middle East* OR South Africa OR Roma OR Muslim OR Buddhist OR Hindu	National origins or cultures underrepresented in the literature
	**Setting**	
5	Autis* OR Autis* Identit* OR ASD OR Autism Spectrum Disorder	Autism
6	Qual* OR interview OR experience OR perception	Individual’s experiences/perspectives
	**Implementation**	
7	1 OR 2 OR 3 OR 4	Culturally and ethnically underrepresented
1	5 AND 6 AND 7	Individual’s experiences/perspectives in underrepresented cultural and ethnic populations

### Screening

**Fig 1:**
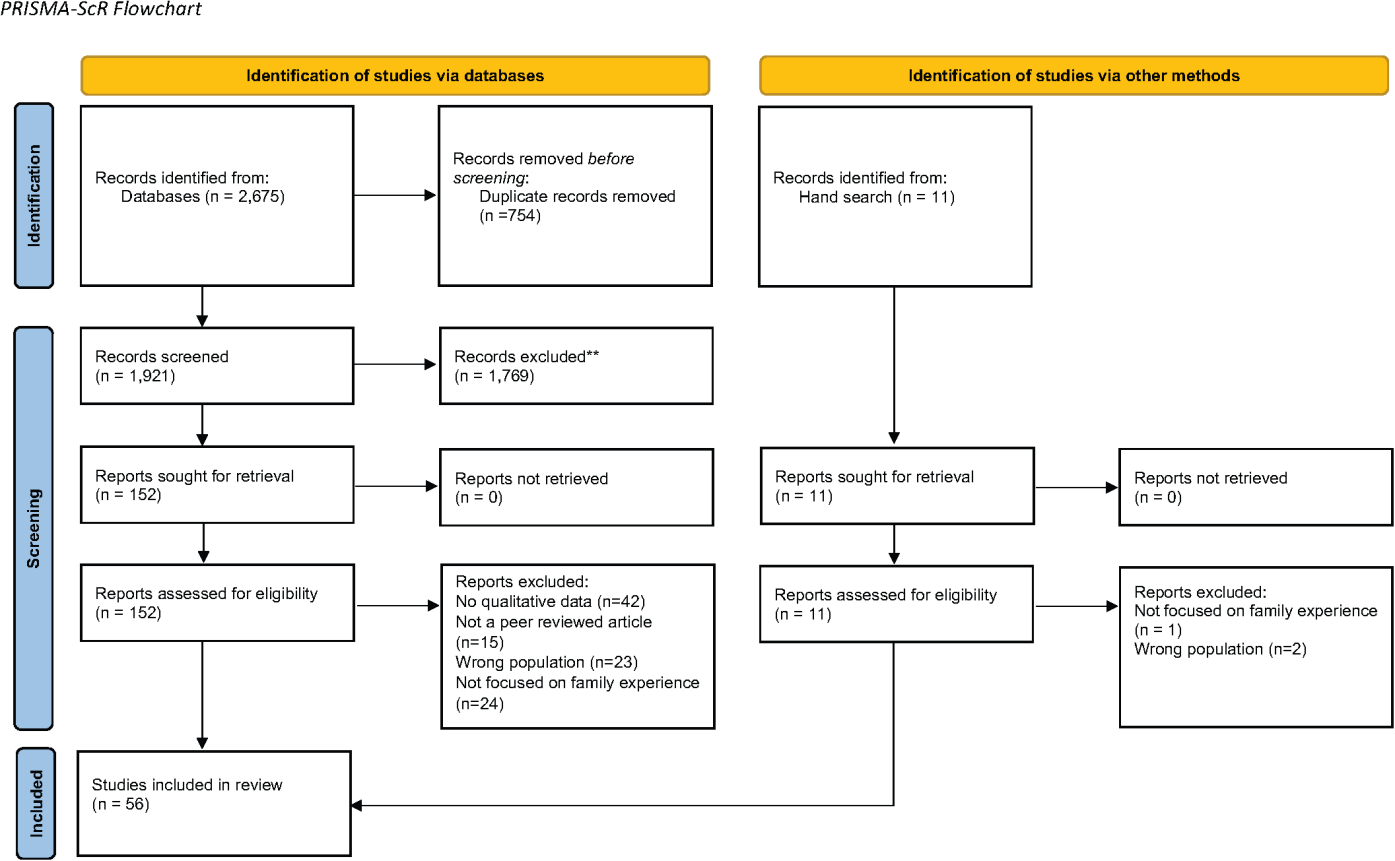
PRISMA Diagram.

Aligned with JBI methodology, three review phases were completed including title and abstract screening, full-text screening, and data extraction. For each phase, two authors independently completed the work in Covidence [32], with disagreements resolved through discussion with a third member. The two reviewers first completed a title and abstract screening, followed by a full-text review. See Fig 1 for PRISMA diagram.

### Data extraction

Data from each study were extracted in Covidence by one of two authors and collated in an Excel spreadsheet. Characteristic data (author name, year, country, sample size, methodology, and population) and outcome data (themes and subthemes emerging from qualitative data) were entered into this spreadsheet. Extracted characteristic data are presented in S1 Table. A calibration exercise was performed prior to full data extraction to ensure that extraction was consistent between authors. Inter-rater agreement during the calibration exercise was excellent (92%) and only minor clarifications were needed before beginning full extraction.

### Data synthesis

Data synthesis was guided by qualitative content analysis [33] and aimed to capture nuanced intersections of race and disability, avoiding reductive analyses that treat these identities as separate. A priori codes informed by Disability Critical Race Theory [26] (e.g., disability-based stigma, social barriers to inclusion) and inductive codes (e.g., parent emotions, religion) were generated to analyse the extracted data [34]. Codes generated through the lens of Disability Critical Race Theory focused on systemic barriers and the implications of systemic oppression rather than attributing disparities to individual or community deficiencies. For example, codes relating to providers’ dismissal of parental concerns were interpreted as reinforcing racialized stereotypes of minoritized families rather than isolated lapses in practice. These codes were then grouped into content categories, such as “Family understanding of autism” (see S2 Table for example quotations). The team engaged in a discussion of these content categories, which were then brought to the advisory board. Through discussion, the content categories were merged, re-organized, and re-labelled to form the following three themes: 1) “God created this world with different people”: Misunderstandings to Advocacy, 2) “You can tell they look down on us”: Inaccessible and Culturally Inappropriate Services, and 3) “The only Black family in Autism-focused spaces and the only family with an Autistic child in Black spaces”: Disability-based Stigma. For example, “Family understanding of autism” and “Experience of having and Autistic child” were merged into “God created this world with different people”: Misunderstandings to Advocacy. The first two theme names are quotes from participants in included studies, while the third theme name is a paraphrase of a quote from an included study.

### Trustworthiness measures

The PRISMA checklist (see S1 Checklist) was used to guide the reporting of this scoping review, providing a standardized framework for transparency and reproducibility. This approach minimized inconsistencies and ensured comprehensive documentation of all steps in the scoping review process. Inclusion and exclusion criteria were established a priori and rigorously applied. A standardized charting form facilitated the data extraction process. The authors engaged in peer debriefing throughout all phases of the study. To enhance the findings’ relevance and applicability, stakeholder consultations were conducted. This manuscript includes all necessary details to enable readers to assess the feasibility of reproducing the search strategy, modifying search terms, or conducting a more detailed analysis.

## Results

### Characteristics of included studies

The article selection process is described in Fig 1 using the PRISMA flow diagram [30]. Fifty-six studies were included in the review, of which 38 were conducted in the United States, six in Canada, two each in Australia, Israel, New Zealand, Sweden, and the United Kingdom, and one in Ireland. One study recruited participants from Taiwan and the United Kingdom [35].

Studies examined the experiences of Black, Latino, Somali, Chinese, South Asian, Indigenous, Korean, Bedouin, Arab, Burmese, Taiwanese, Traveller, and mixed minoritized ethnic groups. There were no included studies that met the inclusion criteria from Indigenous families in Canada. Included studies were published between 2006 and 2025 and recruited between 3 and 400 participants (m = 39, SD = 54). A total of 1454 participants were recruited across all studies.

The most common participants were parents or caregiving family members of Autistic children, with only two of the studies consulting Autistic children themselves [36,37]. Mothers of Autistic children were the only study participants included in 10 studies, and mothers were participants in the majority in 42 studies.

Most included studies used semi-structured or unstructured interviews for data collection (n = 49), with many lacking identification of their qualitative approach (n = 36). In studies that did identify a qualitative approach, phenomenological (n = 5), ethnographic (n = 4), and grounded theory (n = 4) approaches were most common. Four studies used focus groups, and three studies reported using qualitative survey design.

### Theme 1: “God created this world with different people”: Misunderstandings to Advocacy

This theme addressed how families moved from lack of knowledge about autism to acceptance and advocacy for their Autistic child. In many studies (n = 30), families described having gaps in knowledge about how autism presents in children. This knowledge gap led to concern and then educating themselves about autism. Sources of information about autism varied widely and were often linked to support systems. Parents had varying starting points for understanding of autism, some having never heard of the term, or stating there was no word for autism in their language, presenting a substantial barrier to learning how it might align with their child’s traits [37–47]. A lack of available information or the child’s traits not matching the parent’s understanding of autism commonly led to caregivers denying the existence of autism, refuting the child’s diagnosis, or thinking their child will “recover” or be “cured” [38,40,42–45,48–53].

Access to reliable sources of information enabled caregivers to move from lack of knowledge to expertise [54,55]. Families who sought information and resources were better able to accept and come to understand their child [45,49,51,56–58]. Acceptance of their child’s autism diagnosis ultimately allowed caregivers to improve their relationship with their child, shift their expectations of their child, and set new goals that accounted for the child being Autistic [56,57]. Parents used their expertise of their child to advocate for their rights to reach their full potential and learn skills, as well as to help others understand their child’s neurodivergence [59–62].

Many parents reacted to their child’s initial diagnosis by attempting to understand what had “caused” their child to be Autistic [40,63,64], with some Somali parents searching for supernatural or environmental causes such as a punishment for past wrongs or Jinn [37,64,65]. Parents’ faith that a higher power determined that their family would be a safe environment for a vulnerable child, supported their sense of competence as it indicated their ability as parents [38,64–67]. For example, many Islamic families included in the studies referenced Allah’s plan for their family and the meaning it gave to their difficulties understanding their child [59,65,68]. The faith that their child had been given to them for a reason gave parents strength to work with their child’s needs and to find them a place in their community.

The experience of families in participating studies of their child receiving an autism diagnosis began with coming to terms with the idea that parenting their child will be different than parenting a neurotypical child [69]. Typically, parental emotions directly after diagnosis were negative [39,46,50,51,70,71]. Lack of support such as social connection and support contributed to some parents’ poor emotional well-being [60–62,70,72]. Families experienced challenges due to lack of social support through community and extended family [60,62]. Some families had greater resilience through reframing the diagnosis as an opportunity for growth [39,54] which supported them to educate others with their newfound knowledge [73] and advocate for their child to increase access to services [67,74,75].

Included studies described family life rearranging in response to the Autistic child [39] with the main caring responsibilities falling to the mother or a close female relative [48,50]. While descriptions of a negative impact of the child’s support needs on the family dominated the literature, such as limiting family activities [76], the few mentions of positive impacts of having an Autistic child in the family described increased family cohesion in immigrant families [39,51,76], increased religious faith [51], and becoming free of caring about other’s opinions about their family [47].

### Theme 2: “You can tell they look down on us”: Inaccessible and Culturally Inappropriate Services

Issues with accessing appropriate services were evident across over half of the included studies (n = 31). Participants in many studies found the diagnosis and services pathway to be unclear [41,42,45,49,67,77,78] and that services accessed did not fulfil their care needs [41–47,49,53,58,62,70,78]. Participants described challenges regarding providers not understanding autism [45,47,49], poor quality of services [43,47], culturally inaccessible services [41,46,53], and lack of access to services due to cost, availability, or distance [41–43,46,47,58]. Caregivers reported their children being evaluated using culturally inappropriate tools, leading to mistrust of the diagnosis [41,46]. Caregivers in included studies experienced a disconnect between the developmental and social expectations in their own culture and those of the healthcare providers [40,61,79,80]. For example, one study illuminated how Chinese families and Western schools experienced differences in how they engaged in routines related to voice volume, biggest meal of the day, and dental hygiene [50]. Similarly, in another study, [53]providers were found to value social skills and activity of daily living development while families valued academic achievement as they considered activities of daily living as a family responsibility [81]. Additionally, Diné/Navajo families described how Indigenous ways of supporting children were perceived inadequate as compared to formalised therapies and services by providers [43]. When healthcare providers did not respect family’s parenting decisions and discipline practices, this hindered rapport and trust [66,81].

There was emphasis within included articles on the importance of the family-provider relationships, which required communication and trust [45,63,80,82] with a poor relationship leading to culturally inappropriate services [63,70,75,79,81]. Language barriers were common for immigrant families in English speaking countries [37,40,42,44,51,53,62,63,80,83], including use of interpreters with the incorrect dialects for family needs [46], leading some participants to seek services from non-medical professionals with more flexibility and availability in their home languages [84]. English speakers also experienced access challenges with services due to providers’ use of jargon [42,45,78]. Families from non-English speaking backgrounds felt their concerns for their child were dismissed due to their lack of English proficiency [62,85]. Some families from non-English speaking backgrounds were reluctant to engage with services, believing that intervention in a language not used in their home would have negative impacts on their child’s development [83], while other families were told not to speak their home language, even when they did not have conversational skills in English [40,44]. In contrast, when supported by family navigators, immigrant mothers experienced greater engagement and satisfaction with the healthcare system [79].

Racially and ethnically minoritized study participants recounted that service providers treated them differently than white families. Service providers talked to them in an infantilising or condescending manner, did not believe them, ignored them, stereotyped their children, and tried to pacify them rather than take their concerns seriously [36,41,47,49,52,58,66,74,51,78,86]. Families perceived that resources were directed more towards white communities [19,41,43,52,67,74,75]. Black families described how they had to fight harder to access services [19,41,60,74], and dialect switch to be taken seriously [87], and wished that there were providers that looked like them and understood their cultural background [41,78].

### Theme 3: “The only Black family in Autism-focused spaces and the only family with an Autistic child in Black spaces”: Disability-based Stigma

This third theme explores how families had differing experiences of community connection and their actions were often driven by fear of disability-based stigma (n = 32). Due to feelings of shame and embarrassment brought on my outsider’s perceptions, and to protect their Autistic child, some families kept their child’s diagnosis secret or did not bring them out in public [42,53,61,62,72]. Other families described how they did not hide their child, but other parents of Autistic children within their community did [59,61,86]. Disability-based stigma was experienced in judgement from others [40–42,44,46,47,53,62] likely due to misperceptions and a lack of understanding about autism in their community [51,57,67,71,74,86]. Korean mothers described how the cultural value of fitting in and “saving face” was at odds with their child’s behaviours, impacting their family reputation [51,71]. Black participants often found themselves the only Black family in Autism-focussed spaces, and the only family with an Autistic child in Black spaces [52,58,60,74,78]. Families valued the times that they were able to connect to other families of Autistic children from their culture [51,53,80].

Families in many studies described the intersection of racism and disability-based stigma impacting their daily lives, including exclusion from school settings and inadequate resources to support Autistic children and their families [47,60,62,66,75,86]. Families perceived that racism and disability-based stigma compounded their challenges [87], and they wished that providers and community members better understood what Autistic traits looked like in Black children [41]. Black and Latino caregivers worried that this intersection threatened their child’s safety due to risk for abuse in healthcare settings or negative interactions with law enforcement [50,60,75,78,87–89].

While a lack of community awareness of autism was common [37,65,70] some communities were reported as being supportive of the child’s differences by accepting who the child is and nurturing them [43,63,66,80,87,90]. Community support allowed caregivers to better advocate for their child and to support their inclusion [46,48,56,66].

## Discussion

Participants across this scoping study experienced challenges with learning about autism, accessing services that aligned to their values, and feeling welcomed within their cultural community and the larger community due to disability-based stigma and racism. The current study had similar findings to systematic reviews and scoping studies that included studies with predominately white families of Autistic children, such as challenges with learning about autism and adapting to having an Autistic child [91–93], communicating with professionals [91,92,94], and addressing stigma [92]. However, families in the current study also experienced intersectional challenges related to ableism and racism, and unique aspects of growth through leveraging their cultural capital to engage in advocacy. While participants in the Legg and Tickle (2019) study were dismissed by providers for having concerns that their child may be Autistic, and families in the Boshoff et al. (2021) study experienced providers not understanding how to interact with or accommodate for their Autistic child, their legitimate challenges with ableism were not compounded by experiences of racism or lack of culturally or linguistically appropriate care. This intersectionality is the unique contribution of the current study.

As with many scoping studies, the data are, by nature, heterogenous. We sought to synthesize the available data on this topic, identifying common themes across different groups of people and communities. Similar to previous scoping reviews addressing the needs of Aboriginal Australians and Māori peoples, the current review identified challenges with families understanding an autism diagnosis, accessing culturally attuned services, and stigma within the community [24,25]. The current review adds nuance to the challenges faced by families, a broader scope of included articles, considerations of intersectionality, and as well as a synthesis of the current literature. The findings can inform the supports offered to families by educators, health and social care professionals, and disability advocacy and community groups.

## Clinical implications

Disability Critical Race Theory [26] argues that many clinical practices are rooted in racialised and ableist conceptualisations of typical development [95]. This disadvantages racially and ethnically minoritized Autistic children as the support offered neither considers their cultural backgrounds nor appropriately supports inclusion in their communities. It is common for Autistic children to be treated as less able than their more privileged counterparts and with their rights unmet, they are unable to fully participate within their community [96]. From the findings of this scoping review several implications to practice are discussed below using the lens of Disability Critical Race Theory. The potential implications explore strategies that can be used to support racially and culturally affirming paediatric clinical practice for Autistic children.

### “God created this world with different people”: Misunderstandings to Advocacy

The scoping study identified a lack of understanding of autism prior to diagnosis by racially and ethnically minoritized families. Much of the information parents did have access to reflected negative perceptions of autism [39,70]. This overall lack of understanding reflects a challenge as well as an opportunity for the first information accessed to reflect neurodiversity-affirming understandings of autism, and the priorities of the Autistic community. Education should be culturally relevant and empowering, making families first encounter with learning about autism a positive one. For example, providers can help families to use their spiritual capital to cope with stigma, and their aspirational capital to explore their dreams for the future [97]. This can support families to leverage their post-diagnostic growth to engage in advocacy and focus on flourishing as a family [97]. Some culturally validated programmes such as FACES and Parents Taking Action that have been shown to support Black and Latino families in increasing their autism knowledge, self-efficacy, and advocacy skills [98,99]. However, in direct contrast to neurodiversity affirming approaches, these programs frame autism using a behavioural and impairment-focused lens, support families to advocate for behavioural services such as ABA [87], and teach families to address perceived deficits such a social skills and behavioural differences. While advocacy is crucial for families of Autistic children, especially from racially and ethnically minoritized backgrounds, advocacy must be intersectional, considering both what is valued within the families’ culture as well as within the disability community.

Provider bias can lead to incorrect or missed autism diagnoses in racially and ethnically minoritized children [97,100]. Some participants in this scoping study advocated for their children to be referred for an autism diagnosis despite provider reluctance, while others were unaware that their child may be Autistic prior to a referral. Late diagnosis can further intersect with systemic racism, causing racially and ethnically minoritized Autistic children to be more likely to be suspended from school or have other disciplinary actions against them [101]. Black and Latino families in this scoping study were especially fearful of their law enforcement mis-perceiving their Autistic child’s actions and threating their safety.

Awareness of autism in racially and ethnically minoritized communities must be improved to allow for identification and appropriate support of Autistic children. Practitioners should empower and support parents who think their child may be Autistic to explore resources and services that can nurture their child’s strengths and wellbeing. Additionally, if a practitioner believes a child may be Autistic, they should collaborate with the family to share information and guidance that supports parents in making an informed decision about pursuing a diagnosis. For example, this may involve connecting families with culturally appropriate support groups for parents of children with disabilities. Lack of family and community supports can negatively affect how the diagnosis of autism is received by families and contribute to the burden narrative of having a child with a disability that is present across many cultures [102]. There is a need to shift the blame of stress away from the Autistic children and put the onus onto inaccessible communities and systems.

### “You can tell they look down on us”: Inaccessible and Culturally Inappropriate Services

Participants across studies described lack of access to services as a challenge for supporting their Autistic child and family [37,63,67,70,77,83,85]. Care coordination can support families in navigating health and educational systems, helping them access culturally and linguistically appropriate services and connecting them with additional supports [82]. There is a need for paediatric practice to be neurodiversity-positive, culturally affirming, and financially, geographically, physically, socially, and culturally accessible. Intervention should focus on promoting inclusion in the child’s own community, especially in early life. This can be achieved by adapting cultural events or activities to meet the needs of the Autistic child. Providers can work with the child’s family to discuss valued roles, routines, and activities within their community, to identify barriers to participation, and to adapt the activity to meet the child.

Inequities extend beyond diagnostic and service pathways to mental health outcomes. Evidence suggests that racially minoritized Autistic children and their families face heightened risks of psychological distress, including elevated caregiver stress [60]and concerns about safety related to stigma and law enforcement [103]. Our findings illuminate how structural racism and ableism jointly contribute to these disparities, underscoring the need for culturally responsive, community-based supports that address both service access and mental health wellbeing.

Trust in providers was a challenge for many participants in the included studies [63,80,82]. Families described experiences of culturally inappropriate services and overt racism from providers. Adopting critically reflexive practice can support providers to address injustices in health, social care, and education as they engage in reflexivity on their own biases related to disability, culture, and race [95]. For example, providers can reflect on ways that they perpetuate racism and ableism within their practice through considerations of what is normal, and who is at risk to support the provision of services in a culturally-safe and neurodiversity-affirming manner [95,104]. Additionally, rapport with racially and ethnically minoritized clients can be strengthened by recruiting and retaining more providers from similar racial and ethnic backgrounds [105].

### “The only Black family in Autism-focused spaces and the only family with an Autistic child in Black spaces”: Disability-based Stigma

The presence of disability-based stigma was a common concern across the participants in this scoping study. This finding aligns with the experiences of disability-based stigma against persons with disabilities globally [106]. While disability-based stigma is a common challenge for families with Autistic children, racially and ethnically minoritized families experience disability- based stigma and racism and xenophobia [25,107]. Participants described not fitting into either racially and ethnically minoritized spaces or spaces for caregivers of Autistic children, compounding their sense of isolation. There is a need to direct resources to racially and ethnically minoritized communities to support community-led support for Autistic children and their families, as well as information to the wider community to make autism-focussed spaces more inclusive of racially and ethnically minoritized families. Rather than offer services with a universal approach, service providers should consider the intersectional identities of the participants, as racism and ableism are inextricably linked [100]. For example, a support and social group for families with Autistic children could include events that are aimed towards families with certain intersecting identities such as Somali families with Autistic children, or racially and ethnically minoritized families with Autistic children who also identify as LGBTQ+ to address their unique needs. Community groups should understand community needs prior to offering services to align with community values. For example, Black families may prefer informal support discussion rather than leader-led discussion to align with their cultural norms and values [97].

The racism and disability-based stigma identified in this review also carry significant mental health consequences for Autistic children and their caregivers. Persistent experiences of racism and discrimination in healthcare and education can heighten caregiver stress and contribute to symptoms of anxiety, depression, and trauma [19]. For Black and Latino caregivers in particular, fears regarding their child’s safety in interactions with providers exacerbates chronic stress and psychological distress [103]. Likewise, navigating inaccessible systems or being excluded from culturally appropriate services can leave families feeling isolated, undermining their wellbeing and resilience. Stigma within both community and professional settings not only limits access to support but also deepens emotional burden, reinforcing cycles of distress and exclusion. These intersections illustrate that the inequities described in this review are not solely barriers to service access but are also key drivers of mental health disparities within racially and ethnically minoritized Autistic populations.

Although some participants had a negative experience of having an Autistic child within their communities, it is important to build off the positive experiences identified. For example, community support and inclusion were especially strong within Māori [63] and Diné/Navajo [43] communities. This may be due to the holistic, communal, and relational worldviews inherent within the Indigenous culture [108]. Models of care for families across cultural and ethnic groups that incorporate these Indigenous worldviews would align with neurodiversity-positive practice, and the cultural model of disability.

## Limitations & future research

This scoping study was impacted by limitations based on selection bias. The inclusion criteria of studies being written in the English language inherently excludes any studies written in other languages. This common limitation was practical due to the language limitations of the authors; however, is especially important given the international scope of this study. Some useful studies conducted by researchers in their own culture and own language may have been excluded. The research question lent itself towards studies that were qualitative in approach; however, this excluded systematic and narrative reviews and quantitative studies that may have provided important additional information.

This scoping review provided perspectives from families across many cultures, which is both a strength and a limitation of the research. Immigrant populations in high income countries may differ in characteristics from those who live in their country of birth in their level of education, socioeconomic status, and privilege. We did not differentiate between families who chose to immigrate to a new country and refugee or asylum seekers, Indigenous peoples, and descendants of enslaved peoples who would have differing characteristics. Furthermore, while immigrant, refugee, and Indigenous populations were grouped under the term racially and ethnically minoritized*,* their histories, migration trajectories, and relationships to systemic oppression are distinct, and thus the findings should not be assumed to apply uniformly. We sought to mitigate this limitation by specifying particular groups when discussing individual study findings; however, we acknowledge that synthesizing the data across racially and ethnically minoritized populations may obscure within-group differences and limit the nuance of our analysis. Thus, results are valuable to inform clinicians in the global North working with underrepresented populations but may not be generalizable to needs of similar populations in the global South. Moreover, systemic and policy differences between global North countries, such as the privatized healthcare model in the United States versus the publicly funded National Health Service in the United Kingdom, influence service access, timeliness of diagnosis, and equity of provision. These differences also limit the extent to which our synthesis can capture the full complexity of families’ lived experiences.

Within the current literature, there are no studies that met the inclusion criteria from Indigenous families in Canada, and only one study from the United States of America. Future research should address the current under-representation of Indigenous voices in the literature on their experiences of having an Autistic child. Some Indigenous peoples are understandably reluctant to engage with researchers from Western cultures due to historical and contemporary acts of abuse and genocide against Indigenous cultures. Thus, there is a need for Indigenous researchers to use their knowledge to conduct research that reflects the values and priorities of their culture. For example, a relational worldview offers a valuable and distinct perspective on understanding ways to support Autistic children [8].

There were also no studies focused solely on the perspectives of Autistic children. Consistent with literature on lived experience and children with disabilities [109], most of the included studies reflected the perspectives of mothers of Autistic children. The lack of inclusion of Autistic children in research results in work that may not consider their specific rights, needs, and experiences, contributes to greater marginalization of Autistic children, and limits the diversity of experiences examined within the literature. Future research about racially and ethnically minoritized Autistic children should include the perspectives of children and not solely rely on information provided by caregivers. To increase the inclusion of Autistic children in research, researchers can collaborate with Autistic communities and draw from disability and childhood studies to use inclusive, accessible, and non-ableist methods [110].

## Conclusion

Racially and ethnically minoritized families of Autistic children experience compounded exclusion as a result of intersecting disability-based stigma and racism. It is therefore essential for providers to recognize both the shared and distinct experiences of these children and their families and offer culturally attuned services in a neurodiversity-affirming manner. When families are offered opportunities to learn about autism and access to inclusive services that are culturally, racially, and linguistically appropriate, their ability to advocate for their children increases and a greater sense of community belonging develops.

## Supporting information

S1 Checklist PRISMA Checklist. (DOCX)

S1 Table Characteristics of Included Studies. (DOCX)

S2 Table Themes, Categories, Codes, and Example Quotes. (DOCX)
