# Supplementary material for: Navigating racism, stigma, and autism services: A scoping review of the lived experiences of racially and ethnically minoritized families
Source: PLOS Ment Health. 2025 Nov 13;2(11):e0000481. doi: 10.1371/journal.pmen.0000481 (PMC12798594; doi:10.1371/journal.pmen.0000481)
Supplement: S1 Table — (DOCX) [file pmen.0000481.s002.docx]

**Characteristics of Included Studies**

| **Citation** | **Sample**  **(n = )** | **Methodology** | **Country** | **Population** |
| --- | --- | --- | --- | --- |
| (Angell et al., 2023) | 21 | Ethnography: unstructured interview, observation, review of cultural artifacts | United States of America | Latino family members of Autistic children (mothers, fathers, and grandparents) |
| (Birkin et al., 2008) | 12 | Structured interview | New Zealand | Māori, Pasifika, and South Korean parents and teachers of Autistic children |
| (Blanche et al., 2015) | 15 | Semi-structured interview | United States of America | Latino parents of an Autistic child |
| (Bobadilla, 2024) | 9 | Phenomenal: Semi-structured interview | United States of America | Latino mothers of an autistic child |
| (Burkett et al., 2015) | 24 | Semi-structured interview | United States of America | Black family members of Autistic children (mothers, fathers, aunts/uncles, grandparents, and cousins) |
| (Burkett et al., 2017) | 24 | Semi-structured interview | United States of America | Black family members of Autistic children (mothers, fathers, aunts/uncles, grandparents, and cousins) |
| (Camard et al., 2022) | 13 | Semi-structured interview | Canada | Immigrant families of Autistic children |
| (Castelin et al., 2024) | 31 | Group Level Assessment (participatory research method) | United States of America | Caregivers of Black Autistic children |
| (Coffield et al., 2021) | 21 | Semi-structured interview | United States of America | Latino parents of an Autistic child |
| (Dababnah et al., 2018) | 22 | Semi-structured interview | United States of America | Female caregivers of Black Autistic children |
| (Dababnah et al., 2022) | 22 | Semi-structured interview, parental stress measure | United States of America | Female caregivers of Black Autistic children |
| (DuBay et al., 2018), | 20 | Focus group | United States of America | Latino parents of an Autistic child |
| (Fan & Chen, 2024) | 4 | Exploratory case study: Semi-structured interview | Canada | Chinese immigrant parents and grandparents of Autistic children |
| (Ferguson & Vigil, 2019) | 35 | Semi-structured interview | United States of America | Latino and non-Latino parents of an Autistic child |
| (Fong et al., 2023) | 12 | Semi-structured interview | Canada | Korean immigrant parents of Autistic children |
| (Fox et al., 2017) | 15 | Semi-structured interview | United Kingdom | Somali immigrant parents of an Autistic child |
| (Gray & Donnelly, 2013) | 4 | Semi-structured interview | Ireland | Autistic children and their Mothers; Traveller and Non-Traveller communities |
| (Habayeb et al., 2020) | 20 | Semi-structured interview | United States of America | Arab immigrant caregivers of an Autistic child |
| (Huang & Zhou, 2016) | 10 | Semi-structured interview | United States of America | Chinese immigrant parent of an Autistic child |
| (Hussein et al., 2019) | 32 | Semi-structured interview | United Kingdom | Somali immigrant parents of an Autistic child |
| (Ijalba, 2016) | 22 | Semi-structured interview | United States of America | Latino mothers of an Autistic child |
| (Jegatheesan, Fowler, et al., 2010) | 3 | Ethnographic qualitative interview | United States of America | South Asian Muslim immigrant parents of an Autistic child |
| (Jegatheesan, Miller, et al., 2010) | 3 | Ethnographic qualitative interview | United States of America | South Asian Muslim immigrant parents of an Autistic child |
| (Jellinek-Russo et al., 2025) | 4 | Focus group | United States of America | Latina mothers of Autistic children |
| (Kediye et al., 2009) | 10 | Focus groups | Canada | Somali mothers of Autistic children |
| (Kim et al., 2023) | 11 | Semi-structured interview | United States of America | Korean immigrant mothers of Autistic children |
| (Kim & Dodds, 2024) | 10 | Semi-structured interview | United States of America | Korean immigrant mothers of Autistic children |
| (Kizildag et al., 2023) | 26 | Semi-structured interview | United States of America | Immigrant and non-immigrant mothers of Autistic children |
| (Lewis et al., 2022) | 22 | Semi-structured interview, Ways of Coping Questionnaire | United States of America | Female caregivers of Black Autistic children |
| (Lilley et al., 2020) | 12 | Semi-structured interview | Australia | Aboriginal and Torres Strait Islander parents of an Autistic child |
| (Lindly et al., 2023) | 15 | Semi-structured interview | United States of America | Navajo (Diné) parents of Autistic children |
| (Lobar, 2014) | 14 | Ethnographic qualitative interview | United States of America | Latino parents of Autistic children |
| (Lovelace et al., 2018) | 3 | Semi-structured interview | United States of America | Black caregivers of Autistic children |
| (Magaña & Smith, 2006) | 108 | Semi-structured interview | United States of America | Latina and Non-Latina White mothers of Autistic children |
| (Mangum et al., 2025) |  | Semi-structured interview | United States of America | Black mother of Autistic children |
| (Manor-Binyamini & Shoshana, 2018) | 18 | Phenomenological interview | Israel | Bedouin mothers of Autistic children |
| (Manor-Binyamini, 2019) | 19 | Phenomenological interview | Israel | Bedouin fathers of Autistic children |
| (Miller-Gairy & Mofya, 2015) | 25 | Focus groups, participatory observations, and semi-structured interviews | United States of America | Somali refugee mothers |
| (Modirrousta & Harris, 2024) | 4 | Semi-structured interview | United States of America | Three Black mothers of Autistic children and one Asian mother an Autistic child |
| (Nilses et al., 2019) | 11 | Semi-structured interview | Sweden | African, Middle Eastern, and Eastern European immigrant parents of an Autistic child |
| (Onovbiona et al., 2023) | 20 | Semi-structured interview | United States of America | Black caregivers of Autistic children |
| (J. Pearson & Meadan, 2018) | 11 | Semi-structured interview | United States of America | Black caregivers of Autistic children |
| (J. Pearson et al., 2024) | 6 | Semi-structured interview | United States of America | Black caregivers of Autistic children |
| (Pondé et al., 2019) | 44 | Semi-structured interview | Canada | Immigrant parents of an Autistic child from minoritised ethic groups |
| (Rea et al., 2024) | 29 | Semi-structured interview | United States of America | Parents of Autistic children from minoritised ethnic groups |
| (Sakai et al., 2019) | 16 | Semi-structured interview | United States of America | Chinese immigrant parents of Autistic children |
| (Searing et al., 2015) | 92 | Qualitative survey | New Zealand | Māori and Non-Māori caregivers of Autistic children |
| (Shafi et al., 2024) | 9 | Semi-structured interview | Canada | South Asian immigrant parents of Autistic children |
| (Smith et al., 2023) | 15 | Semi-structured interview | Australia | Somali mothers of Autistic children |
| (Thang et al., 2025) | 7 | Semi-structured interview | United States of America | Burmese immigrant caregivers of Autistic children |
| (Tsai et al., 2018), | 14 | Phenomenological interview | Taiwan and United Kingdom | Taiwanese siblings of Autistic children |
| (Weitlauf et al., 2024) | 400 | Qualitative component of survey | United States of America | Black parents of Autistic children |
| (Yang & Crehan, 2023) | 10 | Semi-structured interview | United States of America | Chinese American parents of Autistic children |
| (Yates Flanagan et al., 2024) | 43 | Qualitative component of survey | United States of America | Black caregivers of Autistic children |
| (Yu, 2013) | 15 | Phenomenological interview | United States of America | Chinese immigrant parents of Autistic children |
| (Zakirova-Engstrand et al., 2020) | 17 | Ecocultural Family Interview | Sweden | African, East Asian, European, South Asian, and South American immigrant caregivers of Autistic children |
