# Supplementary material for: Navigating racism, stigma, and autism services: A scoping review of the lived experiences of racially and ethnically minoritized families
Source: PLOS Ment Health. 2025 Nov 13;2(11):e0000481. doi: 10.1371/journal.pmen.0000481 (PMC12798594; doi:10.1371/journal.pmen.0000481)
Supplement: S2 Table — (DOCX) [file pmen.0000481.s003.docx]

Themes, Categories, Codes, and Example Quotes

| Theme | Categories | Codes | Example quotes |
| --- | --- | --- | --- |
| “God created this world with different people”: Misunderstandings to Advocacy | Family understanding of autism  Experience of having an autistic child | Autism perceived causes  Religion  Lack of understanding  Understanding is important  Denial  Acceptance  Advocacy  Experience of obtaining the diagnosis  Parent emotions  Growth  Family life  Finances | “Well, it’s like when you are Muslim, you have to believe whatever happens to you that it’s not coming from you. It’s not your fault. It’s what Allah already wrote and then you have to accept. So it’s the child’s difference. (PPT 2).” (Fox et al., 2017, P.310)  “Knowledge and understanding are so helpful and encourage us as parents to struggle on whereas judgment is so harsh.” (Searing et al., 2015, P.3698)”  “We told them and my dad said, “Why do they say that he has autism? He’s perfectly fine, there is no defect, he wasn’t born with anything defective, he wasn’t born bad.” It’s like I say, we had another idea of what autism was.” (Coffield et al., 2021)  “It was both him and us that had to … agree, we had to understand each other. And I … sometimes he's caught by something … some activity, and doesn't want to leave. But I know now I'm going to have to wait for him. […] … otherwise he's not gonna cooperate. And now it deﬁnitely takes less time than before. (5)” (Nilses et al., 2019, P. 3404) |
| You can tell they look down on us”: Inaccessible and Culturally Inappropriate Services | Service access | Insufficient services  Language barriers  Importance of relationship with provider  Process of intervention  Factors related to using services  Provider racism | “You assume because I’m Black.. . that I might not be as smart. So, I surprise you when I give you all the information. Maybe I was taking it the wrong way.. ..I would get this whole lip service with the doctor, ‘‘Well, you can’t do this with Medicaid.’’ I said, ‘‘I don’t even have Medicaid, I have private insurance.’’...There are assumptions like...I’m just this person that has multiple kids. This is a Black woman. If you see my three kids, you assume I don’t have a husband.” (Dababnah et al., 2018, P329)  “I swear, there is no doctor that I trust. They treat you like you are a child because you come from different part of the world and don’t speak English they way English speakers do, they think that we are not worthy to be informed what they are giving to our children….”(Kediye et al., 2009, P.217)  ‘‘All those years, I kept searching for an appropriate placement for him. I went to the Ministry of Education, supervision. I found a Jewish special education kindergarten. My son was separated from his language, culture, roots. There was no other choice.” (Manor-Binyamini, 2019, P. 7) |
| “The only Black family in Autism-focused spaces and the only family with an Autistic child in Black spaces” | Perception of child in the community | Disability-based stigma  Nurtured/embraced  Neutral  Lack of awareness  Isolation | “Give them [the children] a place in society . . . because on a daily basis we keep facing judgemental and accusatory looks [. . .] in an ideal world, it is first about changing people’s mindsets and also not to bring them [the children] to become neurotypicals, to accept them the way they are, the way they really are.” (Camard et al., 2022, P. 7)  “We have a community center for the Arab community. We get together at least once a month. Anytime he would scream or be sitting there playing by himself, there's looks and there’s whispers. It kind of kept us out of the community for a while, when it was first happening. I kind of distanced myself a little bit.” ((Habayeb et al., 2020, P.58) |
